# Antibacterial and biofilm-inhibiting cotton fabrics decorated with copper nanoparticles grown on graphene nanosheets

**DOI:** 10.1038/s41598-023-38723-4

**Published:** 2023-07-24

**Authors:** Jiwon Kim, Seung Hyun Kang, Yonghyun Choi, Wonjae Lee, Nayeong Kim, Masayoshi Tanaka, Shink Hyuk Kang, Jonghoon Choi

**Affiliations:** 1 School of Integrative Engineering, Chung-Ang University, Seoul, 06974 Republic of Korea; 2 Department of Chemical and Biomolecular Engineering, University of Pennsylvania, Philadelphia, PA 19104 USA; 3 Department of Plastic and Reconstructive Surgery, Chung-Ang University Hospital, Chung-Ang University College of Medicine, Seoul, 06973 Republic of Korea; 4 Feynman Institute of Technology, Nanomedicine Corporation, Seoul, 06974 Republic of Korea; 5 Department of Chemical Science and Engineering, Tokyo Institute of Technology, 4259 Nagatsuta-cho, Midori-ku, Yokohama-shi, Kanagawa 226-8503 Japan

**Keywords:** Nanobiotechnology, Biomaterials, Biotechnology, Nanoscience and technology

## Abstract

Infectious pathogens can be transmitted through textiles. Therefore, additional efforts are needed to develop functional fabrics containing antimicrobial substances to prevent the growth of antibiotic-resistant bacteria and their biofilms. Here, we developed a cotton fabric coated with reduced graphene oxide (rGO) and copper nanoparticles (Cu NPs), which possessed hydrophobic, antimicrobial, and anti-biofilm properties. Once the graphene oxide was dip-coated on a cellulose cotton fabric, Cu NPs were synthesized using a chemical reduction method to fabricate an rGO/Cu fabric, which was analyzed through FE-SEM, EDS, and ICP-MS. The results of our colony-forming unit assays indicated that the rGO/Cu fabric possessed high antibacterial and anti-biofilm properties against *Escherichia coli, Pseudomonas aeruginosa, Staphylococcus epidermidis*, *Corynebacterium xerosis,* and *Micrococcus luteus*. Particularly, the fabric could inhibit the growth of *E. coli, C. xerosis,* and *M. luteus* with a 99% efficiency. Furthermore, our findings confirmed that the same concentrations of rGO/Cu had no cytotoxic effects against CCD-986Sk and Human Dermal Fibroblast (HDF), human skin cells, and NIH/3T3, a mouse skin cell. The developed rGO/Cu fabric thus exhibited promising applicability as a cotton material that can maintain hygienic conditions by preventing the propagation of various bacteria and sufficiently suppressing biofilm formation while also being harmless to the human body.

## Introduction

Cotton fabrics are the most widely used woven textiles due to their sweat absorption abilities and excellent biodegradability, softness, and regenerative capabilities^1^. Cotton is widely used to fabricate various items, including sports products, non-implantable medical products, and healthcare and hygienic products^2^.

In the context of pathogen transmission, fabric is a key medium for infection by various pathogenic microorganisms such as bacteria, fungi, and viruses^3^. Moreover, due to their high hygroscopicity, cotton fabrics are susceptible to microbial attacks^4^. The infection of cotton fabrics by microbes such as bacteria and mold may lead to odor issues and the destruction of the fabric structure. Additionally, because pathogens can be transmitted to humans through clothing and threaten human health, the development of sanitary antimicrobial fabrics is essential to prevent the spread of infection^5^. Antimicrobial fabrics can thus prevent the spread of infections to promote human health and hygiene.

To impart antimicrobial properties to the fabric, reduced graphene oxide (rGO) sheets and copper nanoparticles (Cu NPs) were coated on the surface of cotton fabric. In Cu NPs, ions are released through a redox process and copper ions exert an antibacterial effect by inhibiting the production of enzymes and proteins that bind to DNA^6,7^. Additionally, copper ions exhibit a high affinity for amines and carboxyl groups on the cell surface, which enables them to readily bind to the cells. After entering the cells, these groups induce cross-linking between the nucleic acid strands to destroy the DNA helical structure^8^. Cu NPs are widely used in films, fabrics, metal alloys, and hydrogels, among other applications, due to their antibacterial properties induced by the aforementioned mechanism^9,10^. The multifunctional properties of graphene, such as electroconductivity, UV shielding, hydrophobicity, antimicrobial activity, and biocompatibility, can be shifted to the fabrics embedded with graphene derivatives^11,12^. Especially, GO and rGO make it difficult for bacteria to attach to the fabric because of their rough surface and oxidative stress, in addition to damaging the bacterial cells through the generation of reactive oxygen species (ROS)^13,14^. Notably, in the case of fabrics that are meant to be in direct contact with human skin, non-toxicity or biocompatibility to the host cells must also be ensured in addition to their antibacterial effects. Unlike other metal ions, copper ions not only possess strong antibacterial properties but also show biocompatibility and wound-healing abilities such as stabilizing and regulating the expression of extracellular skin proteins^15–17^.

Recently, many studies have been actively conducted on coating fabrics using Cu or CuO nanoparticles. For example, B Turakhia et al. synthesized copper oxide nanoparticles via green approaches and coated them on cotton fabric to impart an antibacterial effect against various bacteria. The developed material exhibited superior antibacterial effects against *Escherichia coli* even after 30 cycles of washing^18^. Furthermore, Vasantharaj et al. coated green-synthesized CuO nanoparticles on cotton fabric. The authors confirmed antibacterial effects against *Staphylococcus aureus, Escherichia coli,* and *Klebsiella pneumoniae*, and photodegradation effects due to Cu were confirmed. This approach has the advantage of being able to prevent skin irritation and central nervous system poisoning by inhibiting the bioaccumulation of dye^19^. Both studies coated CuO nanoparticles on cotton fabric via the simple dip-coating method. Furthermore, Bhattacharjee et al. fabricated an antibacterial fabric by firmly adsorbing rGO to the fabric using a coupling agent and synthesizing Ag or Cu NPs via the chemical reduction method^11,20^. Antimicrobial activity against *Escherichia coli, Pseudomonas aeruginosa, Staphylococcus aureus,* and *Candida albicans* was maintained even after ten washes, and low cytotoxicity against HEK293 cells was confirmed.

**Scheme 1:**
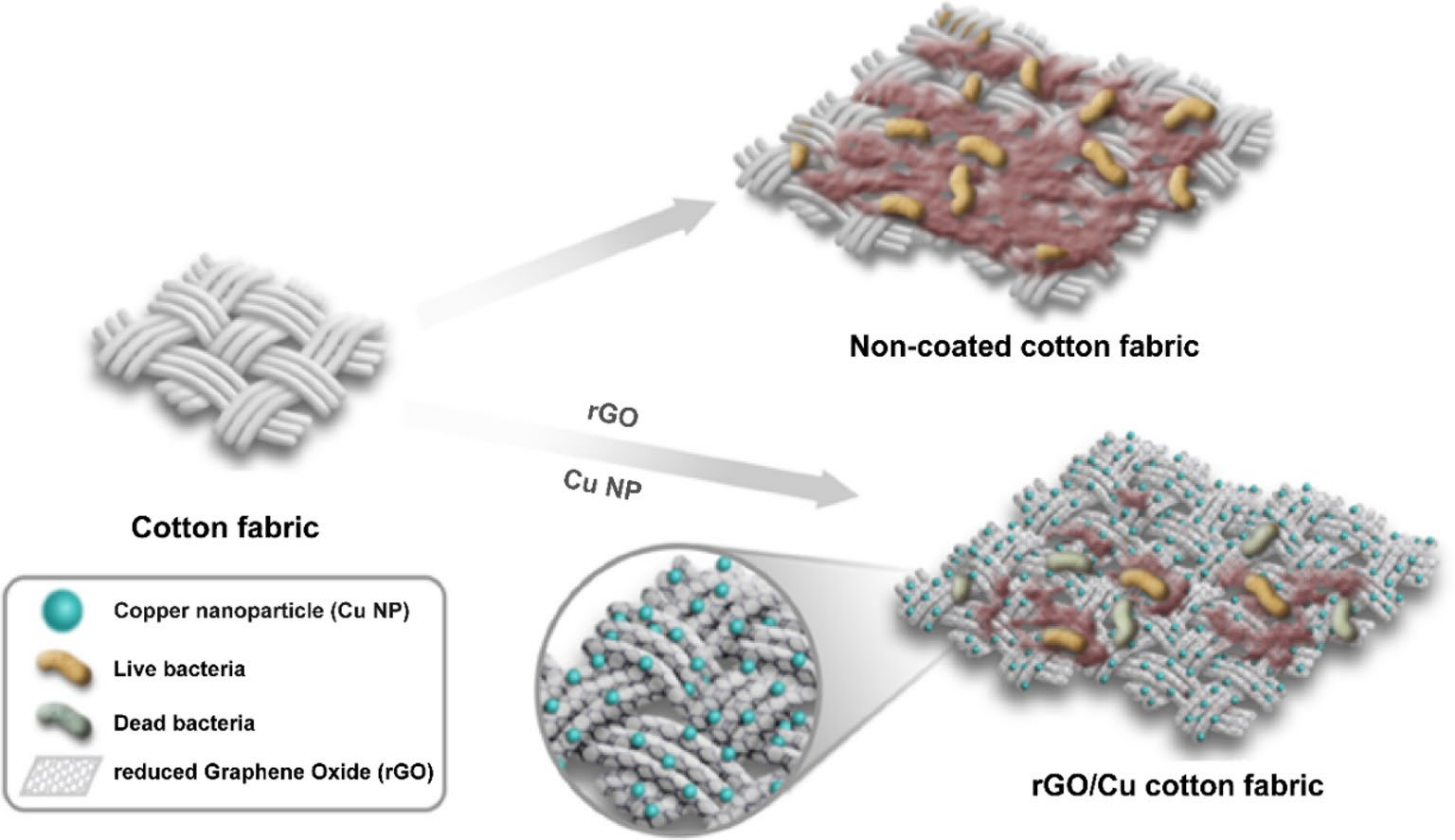
Preparation of antibacterial fabric decorated with rGO/Cu nanocomposites for the prevention of biofilm formation.

In this study, we fabricated disposable fabric that can be used as bandages and surgical gauze that can prevent wound infections or disposable insoles and armpit pads to prevent body odor. To impart an antibacterial effect to the fabric, our study used metal nanocomposites with remarkable antibacterial and antibiofilm effects that we previously characterized^21–24^. rGO was adsorbed onto the fabric via the dip coating method, after which the fabric was coated with Cu NPs via the chemical reduction method. rGO functionalized with Cu exhibits higher antibacterial activity but has lower toxicity to mammalian cells^25,26^. In our previous study, Cu/MWCNTs effectively inhibited the growth of *Methylobacterium* spp. but did not show any cytotoxicity in human dermal fibroblasts (HDFs) even when the same doses were administered. Moreover, Cu suppressed the expression of genes involved in biofilm formation by inhibiting the quorum sensing of *Methylobacterium* spp^21^. In fact, the antibacterial fabrics that we produced had low cytotoxicity to human and mouse skin cells. Furthermore, coating the surface of the fabric with rGO suppresses the attachment of bacteria to the fabric, thus inhibiting biofilm production. In this study, we assessed the antibacterial effects of rGO/Cu NP-coated fabric against *E. coli*, *P. aeruginosa*, *S. epidermidis*, *C. xerosis*, and *M. luteus*. Unlike other studies, our findings confirmed that the rGO/Cu fabric could prevent not only wound infection but also sweat-associated odors by also confirming its antibacterial and antibiofilm effect against *S. epidermidis*, *C. xerosis*, and *M. luteus*, which are known for causing body odor. Bacteria are also notorious for forming strong biofilms, which protect them from the external environment and are difficult to remove even with antibiotics. Therefore, we created a biofilm model for the cotton fabric to confirm that the biofilm could be removed in practice. Our findings confirmed that the rGO/Cu cotton fabric can sufficiently inhibit the biofilm formation of the aforementioned bacteria. To the best of our knowledge, this is the first study to confirm the antimicrobial and anti-biofilm effect of graphene and copper nanoparticles against bacteria causing body odor, especially *C. xerosis*, and *M. luteus*. And rGO/Cu nanocomposites effectively eradicate *C. xerosis*, and *M. luteus*. The antibacterial fabrics produced in this study were confirmed to be highly biocompatible with no signs of cytotoxicity and exhibited remarkable antibacterial effects by inhibiting the production of biofilms (Scheme 1). More importantly, unlike other studies, we compare the antimicrobial and anti-biofilm effects immediately after our rGO/Cu fabric was fabricated and after aeration sufficiently. As a result, our rGO/Cu fabric sufficiently maintained the antimicrobial and anti-biofilm ability.

## Materials and methods

### Materials

GO, sodium borohydride, and dimethyl sulfoxide-d6 were purchased from Sigma-Aldrich (MO, USA). Copper (II) chloride dihydrate was purchased from Reagents Duksan (Ansan, Korea). *Escherichia coli*, *Pseudomonas aeruginosa*, and *Staphylococcus epidermidis* were purchased from ATCC (VA, USA). *Corynebacterium xerosis* and *Micrococcus luteus* were purchased from KCTC (Jeongeup, Korea). CCD-986Sk, HDF, and NIH/3T3 cells were purchased from the Korea Cell Link Bank (Seoul, Korea).

### Fabrication of rGO/Cu fabric

To produce the antibacterial fabrics, 0.25 mg/mL of GO solution and cotton fabric samples (3 × 3 cm^2^) were added and dip-coated at 27 °C with constant stirring for 3 h. After dip coating, the fabric samples were washed with deionized water (DW) until GO residues were no longer detectable. The GO-coated cotton fabrics were immersed in 5 mM CuCl_2_ aqueous solution and stirred for 1 h. Cu^2+^ was sufficiently coupled to the negatively charged functional group of GO through electrostatic attraction. The dip coating process was performed at room temperature. In a 1:1 mixture of CuCl_2_ aqueous solution used for Cu^2+^ coating and 20 mM NaBH_4_ solution, cotton fabrics coated with Cu^2+^ and GO were stirred at room temperature for 30 min to form Cu NPs on the surface of the cotton fabrics and reduce GO. Afterward, to remove the Cu NPs and the remaining NaBH_4_ that was not bound to the cotton fabrics, the samples were placed in DW for 5 min and washed with constant stirring until the leachate reached a pH of 7. Finally, the samples were dried overnight in a 70 °C oven.

Field emission scanning electron microscopy (FE-SEM; SIGMA, Zeiss, Germany) and energy-dispersive X-ray spectroscopy (EDS) were performed to confirm that the synthesized nanoparticles were Cu NPs. The amount of Cu present in the rGO/Cu fabric was measured through inductively coupled plasma-mass spectrometry (ICP-MS; NexION 350D, PerkinElmer, USA).

### Hydrophobicity of rGO/Cu fabric

The hydrophobicity of the untreated fabric and rGO/Cu fabric was measured using a drop shape analyzer (FM40Mk2 EasyDrop, KRUSS GmbH, Germany). A 5 µL DW droplet was dropped onto the untreated and rGO/Cu fabrics. The water contact angle (CA) relative to the fabric surface was measured using the tangent method.

### Cytotoxicity of rGO/Cu fabric

The cytotoxicity of the rGO/Cu fabric to human skin fibroblasts (CCD-986Sk and HDF) and mouse skin cells (NIH-3T3) was examined using the fabric itself and leachate (i.e., the cells were not directly exposed to the fabrics)^5,27^. Fabric was prepared with a concentration of 0.5 cm^2^/mL, after which the cells were treated with the fabric for 24 h to examine the cytotoxicity. Leachate was prepared with a concentration of 0.5 cm^2^/mL, after which the cells were treated with the leachate for 3, 6, 12, and 24 h to examine the changes in cytotoxicity with exposure time. The cultured cells were seeded in a 96-well plate at a concentration of 1 × 10^4^ cells/well with the fabric and leachate for the aforementioned exposure times. Untreated cotton fabric and fabric moistened with 30% DMSO were used as negative and positive controls, respectively. To confirm the cytotoxicity of the rGO/Cu coating, the viabilities of the cells treated with the rGO/Cu fabric and the untreated fabric itself and leachates were compared. For the CCK-8 assay, after 24 h, CCK-8 solution (10% concentration) was added to each well and incubated at 37 °C in a 5% CO_2_ environment for 2 h. The solution was examined at an optical density of 450 nm using a microtiter plate reader (Synergy H1, BioTek, USA). For the Live/Dead assay, after 24 h, calcein-AM (2 μM) and ethidium homodimer-1 (4 μM) were added, and the cells with fabrics were incubated for 30 min at 37 °C. The fluorescence images were taken using a confocal microscope (STRLLARIS 5, Leica, Germany). All images were processed using the Leica microscopes software (LAS X).

### Antibacterial effect of rGO/Cu fabric

Our study confirmed the antibacterial ability of the rGO/Cu fabric against both gram-positive (*S. epidermidis*, *M. luteus*, and *C. xerosis*) and gram-negative (*E. coli* and *P. aeruginosa*) bacteria. Particularly, *M. luteus* and *C. xerosis* are known to cause body odor and were thus used to examine the properties of the antibacterial fabrics coming into contact with human skin. All the bacteria were subcultured in solid and liquid media at 37 °C using a shaking incubator at 24 h intervals. Pure single colonies were isolated to perform the experiment. Similar to the previous cytotoxicity experiment, the fabric treatment concentration was 0.5 cm^2^/mL. The bacteria were inoculated into fabric-soaked media at a concentration of 5 × 10^5^ colony-forming units (CFU)/mL, after which they were incubated for 24 h. The negative control was untreated cotton fabric, and the positive control was cotton fabric moistened with penicillin/streptomycin. The bacterial solution cultured with the sample was diluted to an appropriate concentration, and 100 μL of the bacterial dilution was smeared onto the solid medium to determine the number of colonies. The results were calculated as the viability of the bacteria treated with each sample compared to the viability of the untreated bacteria.

### Inhibitory effect oF rGO/Cu fabric on biofilm formation

The number of bacteria attached to the fabric surface was quantified to confirm the formation of biofilm on the fabric surface and the ability of the rGO/Cu fabric to inhibit biofilm formation. The degree of biofilm formation was visually confirmed through dyeing. The bacteria used for our experiments included *E. coli, P. aeruginosa, S. epidermidis, C. xerosis*, and *M. luteus*. After preparing the fabric leachates at a concentration of 0.5 cm^2^/mL, the bacteria were inoculated at a concentration of 5 × 10^5^ CFu/mL and allowed to incubate for 24 h. The negative control was untreated cotton fabric and the positive control was cotton fabric moistened with penicillin/streptomycin. The fabrics were washed three times to remove non-adherent bacteria. To quantify the number of bacteria attached to the fabrics, the washed fabrics were soaked in sterile water, after which the bacteria attached to the fabrics were removed through vortexing. The bacterial solution cultured with the sample was diluted to an appropriate concentration, and 100 μL of the bacterial dilution was smeared onto the solid medium to determine the number of colonies. The results were calculated as the viability of the bacteria treated with each sample compared to the viability of the untreated bacteria. To examine the biofilm, the washed fabrics were stained with the LIVE/DEAD BacLight Bacterial Viability Kit (Thermo Fischer, USA) and examined using a fluorescence microscope (IF-SERIES, Euromex, Netherlands) with U2-RFLT100 Mercury Power Supply.

## Results and discussion

### rGO/Cu fabric characterization

**Figure 1:**
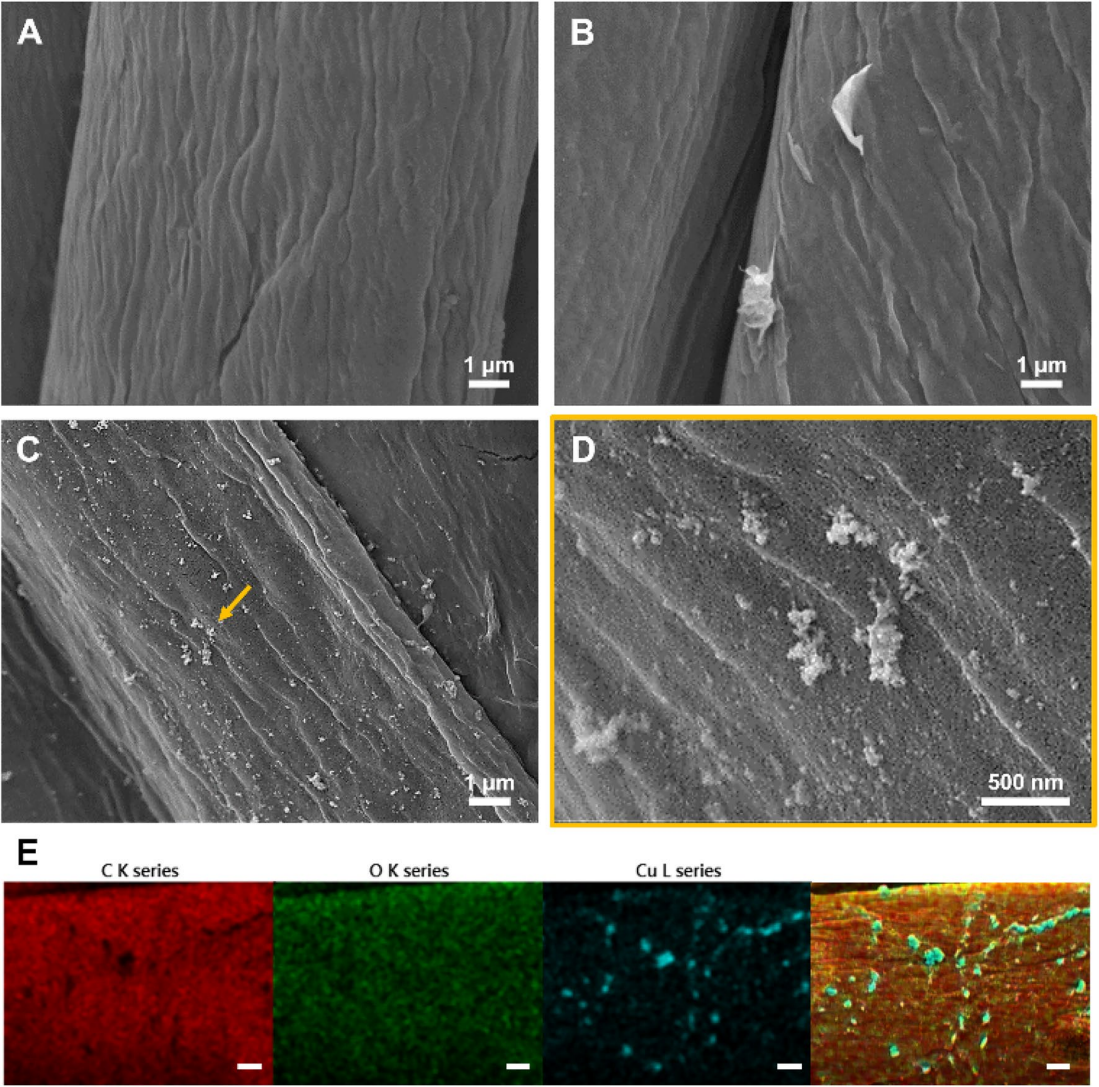
FE-SEM images of (**A**) cotton, (**B**) rGO cotton, and (**C**), (**D**) rGO/Cu fabric (× 10 k), (× 40 k), respectively. (**E**) EDS mapping images of rGO/Cu fabric. The scale bar represents 1 μm.

rGO was adsorbed onto the fabric by dip coating, and Cu NPs were synthesized using the bottom-up method. To confirm that the fabrics were well coated with rGO/Cu, surface analysis was performed through FE-SEM. As shown in Fig. 1, Fig. 1A is the untreated fabric surface, and Fig. 1B is the fabric surface dip-coated with only rGO. rGO was found to be bound to the cellulose surface. Figure 1C shows the image obtained after synthesizing Cu NPs on rGO cotton and Fig. 1D is the magnified view of the region indicated by the arrow in Fig. 1C.

Nanoparticles with an approximate size range of 100–200 nm were synthesized on the surface in an aggregated form. The elemental composition of the samples was examined through EDS. The nanoparticles present on the fabric surface were Cu NPs. In terms of weight (%), C and O accounted for 78.32% and 13.20% of the surface composition, respectively, whereas Cu accounted for 8.49%. Additionally, ICP-MS analysis was performed to quantify the Cu NPs synthesized on the surface of the rGO/Cu fabric. The results demonstrated that an average of 25.44 µg/cm^2^ of Cu was present in the fabrics.

### Hydrophobicity of rGO/Cu fabric

**Figure 2:**
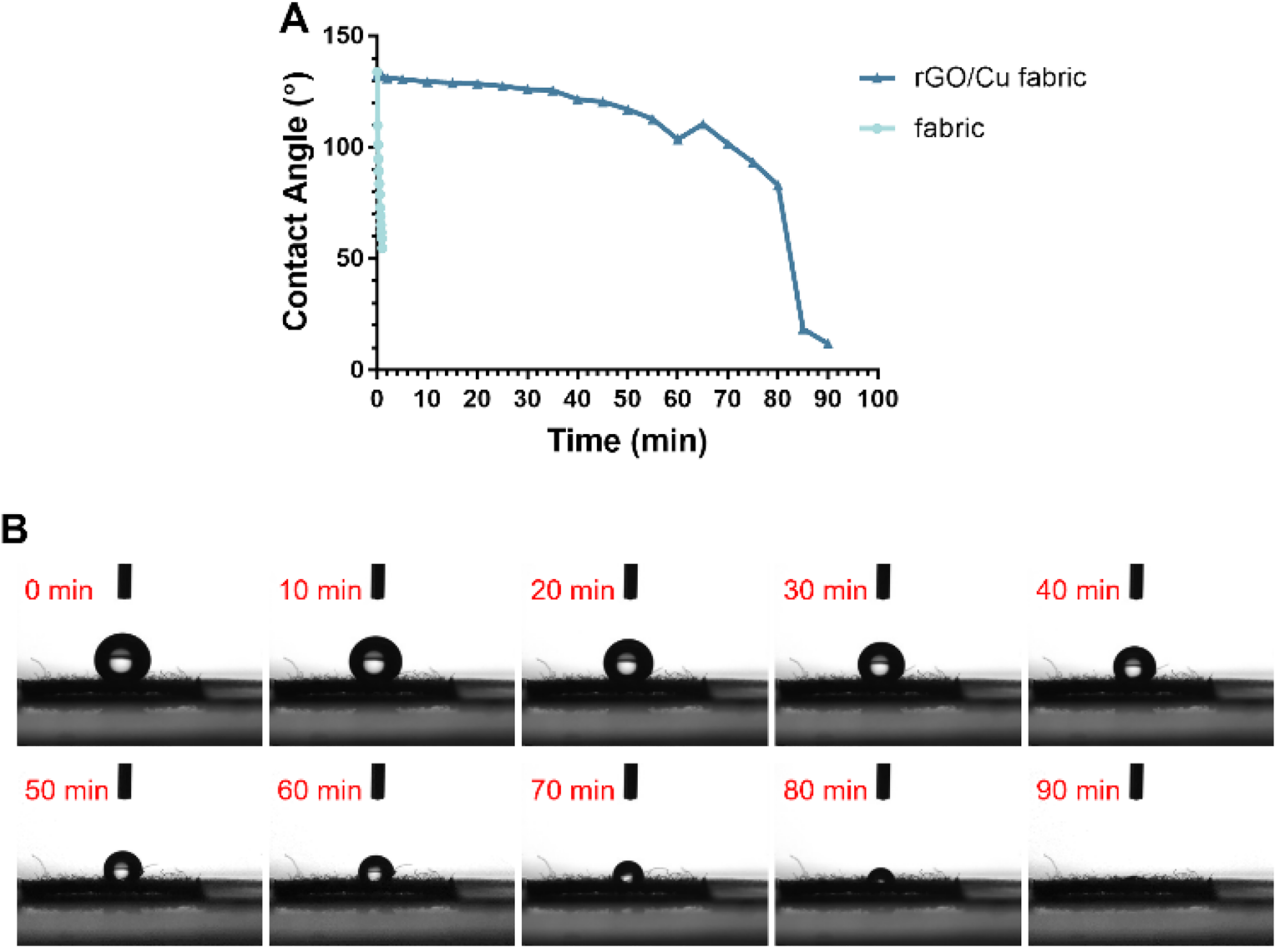
Hydrophobicity test of rGO/Cu fabric. (**A**) Contact angles of fabric and rGO/Cu fabric. (**B**) Images of 5 μL DW droplet on rGO/Cu fabric at different intervals within 90 min.

The contact angle (CA) was measured to confirm the effect of the rGO/Cu coating on the hydrophobicity of the cotton fabric. Initially, as shown in Supplementary Fig. S1, the CA of rGO/Cu fabric was 160.0°, indicating superhydrophobic properties. Therefore, the hydrophobicity was significantly increased with rGO coating on the fabric. To reproduce the properties of an rGO/Cu fabric that would be used in real life, the rGO/Cu fabric was allowed to thoroughly aerate and become re-oxidized for a sufficient time. Figure 2A shows the CA measurement of the DW droplet placed on the fabric at 5 min intervals, whereas Fig. 2B shows a photograph of the droplet on the re-oxidized rGO/Cu fabric at different time intervals. The CA of the untreated fabric decreased to 78.7° in 30 s and decreased further to 54.8° after 1 min (80° lower than the initial value). The rGO/Cu fabrics coated with rGO and Cu NPs exhibited highly improved hydrophobicity, with a CA of over 90° for approximately 80 min.

Cotton fabrics have a large number of hydroxyl groups, and they thus exhibit excellent water absorption properties^28^. Therefore, cotton fabric has a hydrophilic surface that readily absorbs DW. However, in this study, the surface of the fabric became superhydrophobic due to the coating of rGO/Cu on the fabric. While the rGO/Cu fabric was re-oxidized during aeration, its hydrophobicity was lowered but maintained. In general, the hydrophobicity of the fabric surface is determined by the surface roughness and chemical composition^29,30^. The surface roughness of the rGO/Cu fabric was changed by the rGO sheets, and the hydroxyl and carboxyl groups of GO and cotton were removed during the reduction process by NaBH_4_^31,32^. Surfaces with higher hydrophobicity have a stronger antibacterial effect^33^. Particularly, superhydrophobic surfaces have antibacterial adhesion properties, as they naturally possess nano/microscale structures in addition to the repulsion of water, which limits the access of bacteria on the surface^34,35^. Moreover, due to the water repellency, self-cleaning, friction reduction, and antifouling properties of super-hydrophobic coatings, these materials can prevent fabric contamination by inhibiting the absorption of organic pollutants^36^.

### Cytotoxicity of rGO/Cu fabric

**Figure 3:**
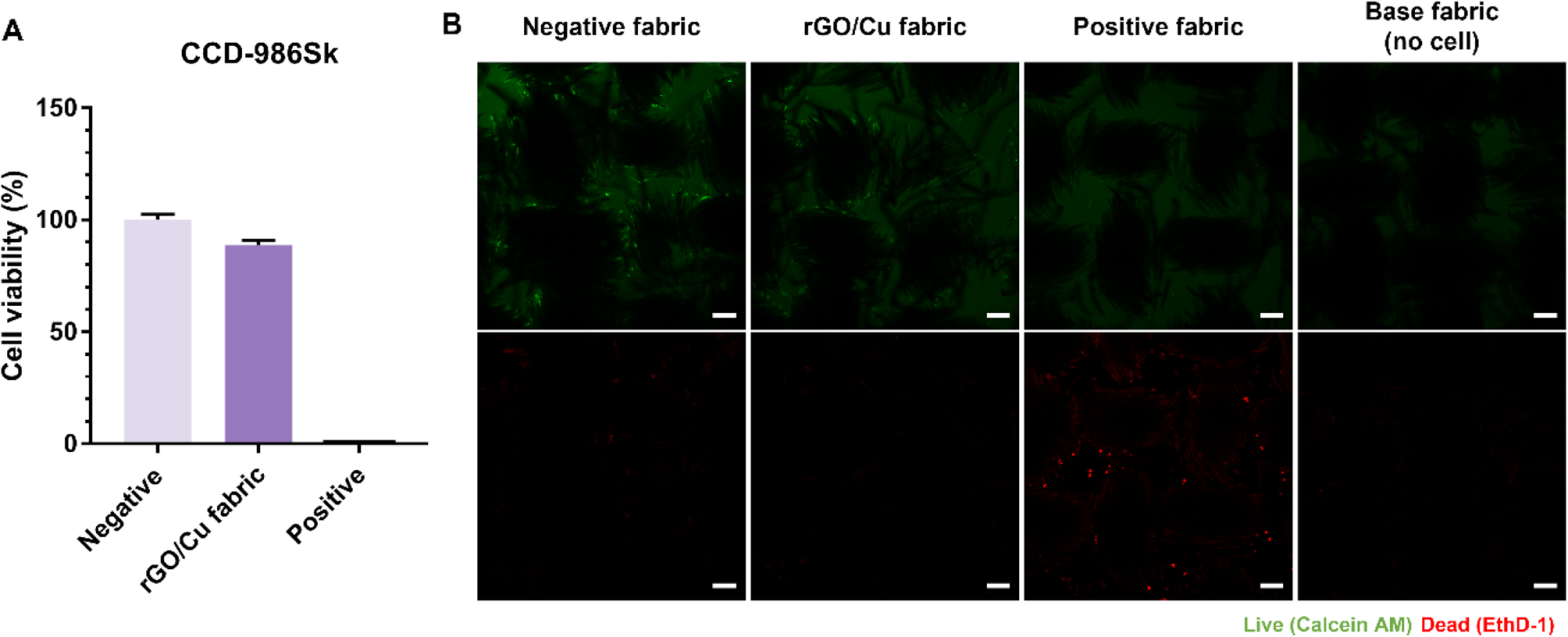
In vitro cell viability test using (**A**) CCK-8 assay and (**B**) Live/Dead assay to CCD-986Sk cells cultured on the raw fabric and rGO/Cu fabric for 24 h. The data are presented as mean ± SD (n = 9).

In addition to bacteria, antibacterial fabrics may also affect normal cells. Given that antibacterial fabrics are expected to directly contact the skin, their cytotoxicity to human skin cell (CCD-986Sk and HDF) and mouse skin cells (NIH/3T3) was examined. Our experiments were conducted to assess the effects of the antibacterial fabrics at exposure durations ranging from 3 to 24 h. Supplementary Fig. S2 shows cytotoxicity when the cells are exposed to leachate of raw fabric and rGO/Cu fabric. As shown in Fig. S2A,B, HDF cells exhibited no signs of cytotoxicity when exposed to the rGO/Cu fabric for 4 h. However, the cell viability decreased to approximately 82% after exposure for more than 8 h. Similarly, the NIH/3T3 cells exhibited no visible signs of cytotoxicity when exposed to the rGO/Cu fabric leachate for 8 h, but their viability decreased to approximately 90% after exposure for more than 20 h. Afterward, the rGO/Cu fabric was allowed to aerate and become re-oxidized for a sufficient time. As shown in Fig. S2C,D, both cell types exhibited higher viability after the rGO/Cu fabric was re-oxidized. Furthermore, the cell morphology was similar to that of the negative control. Also, Fig. 3 shows cytotoxicity when the cells are exposed to rGO/Cu fabric itself. As shown in Fig. 3, 88.6% of cells survived and the Live/Dead images supported that rGO/Cu fabric shows low cytotoxicity.

In general, copper is an important nutrient required to maintain homeostasis. However, if Cu levels exceed the permissible limits by ingestion or inhalation, toxic effects may occur in the respiratory and gastrointestinal tracts^37^. Although cytotoxicity was substantially decreased by re-oxidization, reduced graphene derivatives are known to induce a cytotoxic response by ROS production^38^. However, chemically reduced GO has fewer functional groups than thermally reduced GO. Therefore, its cytotoxic effect and ROS generation potential are low^39^. Several studies have confirmed that rGO has high antibacterial properties coupled with low cytotoxicity^40–42^. Additionally, Maddinedi et al. reported that when 0.05 mg/mL of GO reduced with ammonia hydroxide was used to treat human skin fibroblast cells, the cell viability was 90%^40^. The rGO/Cu fabrics in our study also exhibited extremely low cytotoxicity, confirming that the antibacterial fabrics are biocompatible.

### Antibacterial effect of rGO/Cu fabric

The antibacterial properties of rGO/Cu fabrics were evaluated by quantifying the reduction in the number of CFUs in response to rGO/Cu treatment. Here, we examined the antibacterial effect of the rGO/Cu fabrics on both gram-positive (*S. epidermidis*, *M. luteus*, and *C. xerosis*) and gram-negative (*E. coli* and *P. aeruginosa*) bacteria. Infection with *E. coli, P. aeruginosa,* and *S. epidermidis* through wounds can substantially delay wound healing, which can lead to sepsis in severe cases. Additionally, bacteria such as *S. epidermidis*, *C. xerosis*, and *M. luteus* are known to cause severe body odors upon contact with sweat. Therefore, we investigated whether the developed rGO/Cu fabrics can inhibit the growth of pathogens and odor-causing bacteria.

**Figure 4:**
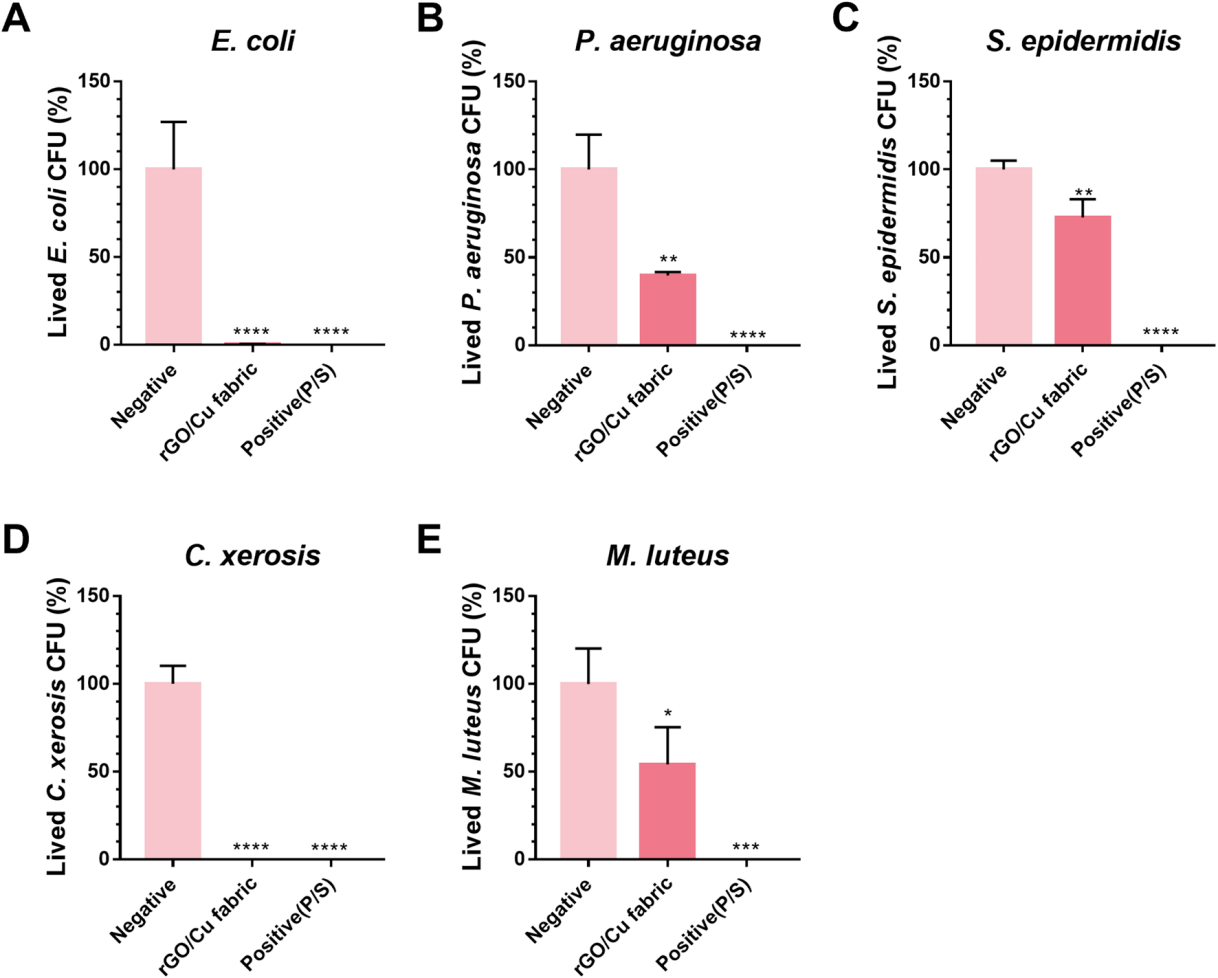
Antibacterial effect of rGO/Cu fabric against (**A**) *E. coli*, (**B**) *P. aeruginosa*, (**C**) *S. epidermidis*, (**D**) *C. xerosis*, and (**E**) *M. luteus*. Untreated cotton and cotton treated with antibiotic–antimycotic (×100) were used as negative and positive controls, respectively. The data are presented as mean ± SEM (n = 3).

After culturing each bacterial with fabrics, the CFU value was counted. As shown in Fig. 4, the rGO/Cu fabrics could effectively inhibit the growth of bacteria. Except for *S. epidermidis*, the fabrics exhibited an antibacterial effect of 50% or more. The growth inhibition rates for *E. coli* and *C. xerosis* were especially high (~ 99.6%). When rGO/Cu is used as an antibacterial material, microorganisms are particularly affected by the rGO and copper ions released by the oxidation–reduction of Cu NPs. Metal nanocomposites were used as an antibacterial material in this study. Metal nanoparticles such as silver (Ag), copper (Cu), zinc (Zn), and titanium (Ti) are commonly used as antibacterial materials^43,52–55^. These materials are known to release metal ions and ROS to cause oxidative damage to the cellular structure. Also they exert an antibacterial effect by not only peroxidizing lipids in cells but also oxidizing proteins and inducing the degradation of DNA^6^. Compared with silver, which is widely used as an antibacterial material, copper ions are cheaper and more stable, in addition to exhibiting higher oxidation resistance^44,45^. Furthermore, copper ions have wound healing properties and are thus considered more promising candidates for producing antibacterial fabrics^17,46^. In our previous study, we fabricated Ag/Cu/GO nanocomposites by synthesizing Ag/Cu bimetallic nanoparticles on graphene oxide nanosheets and confirmed their anti-biofilm effect and wound healing potential in vivo^22^. The wound of the Ag/Cu/GO-treated groups was more effectively closed than that of the control group. These results suggest that Ag/Cu/GO nanocomposites efficiently inhibit biofilm growth and facilitate wound healing. Therefore, as demonstrated by our previous results, the rGO/Cu fabric developed herein is also expected to be beneficial for wound healing, in addition to its antibacterial properties.

### Antibacterial and biofilm inhibition effect of re-oxidized rGO/Cu fabric

**Figure 5:**
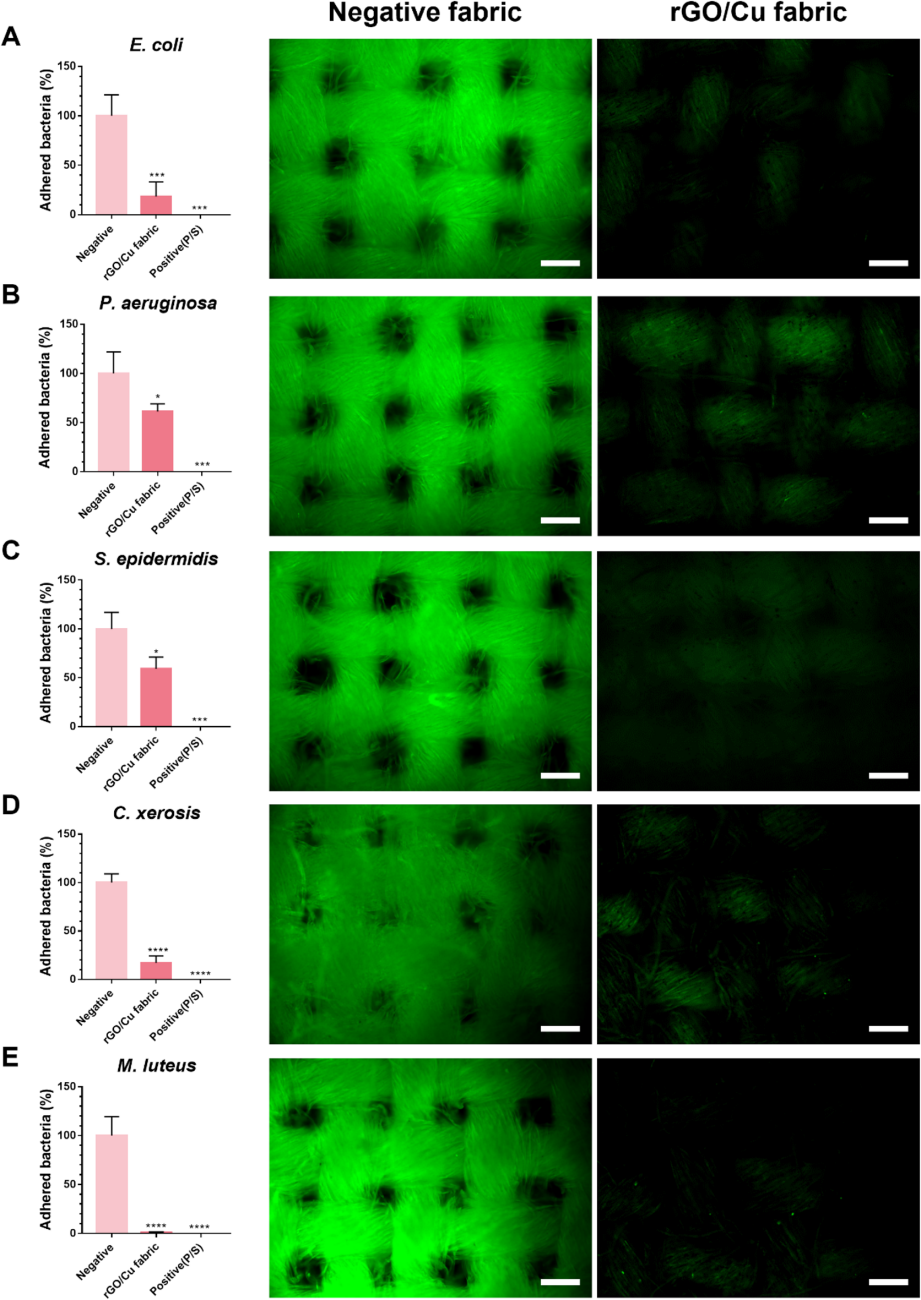
Adhesion and growth of bacteria on cotton and rGO/Cu fabric: (**A**) *E. coli*, (**B**) *P. aeruginosa*, (**C**) *S. epidermidis*, (**D**) *C. xerosis*, and (**E**) *M. luteus*. Fabrics were thoroughly aerated and re-oxidized. The bacteria attached to the fabrics were counted in terms of the CFU to check the concentration of bacteria (CFU/mL). The data are presented as mean ± SD (n = 3). The adhesion was confirmed by staining the bacteria attached to each fabric. The scale bar represents 100 μm.

According to the previous analysis, the fabric surface was super-hydrophobically modified through the coating of rGO/Cu on the cotton fabric. Additionally, our findings confirmed that the rGO/Cu fabric maintained its hydrophobicity even after being thoroughly aerated and re-oxidized. The degree of biofilm formation was examined after 24 h to confirm whether the hydrophobicity of the re-oxidized rGO/Cu fabrics influenced the adhesion of bacteria and biofilm formation (Fig. 5). In general, when bacteria attach to a solid surface, they grow and produce extra polymeric substances (EPSs) composed of polysaccharides, proteins, and glycolipids. As the bacteria grow, the EPS layer accumulates to form a biofilm^47^. A biofilm is a protective film that protects microorganisms from external threats, facilitates communication and metabolism of bacteria present in the membrane, and enables adaptation to any environment. If a biofilm is generated, the bacteria become resistant to antibacterial substances and cannot be easily removed from the fabrics^48^. Particularly, pathogens may adhere to the skin and cause infection and inflammation. In turn, the growth of odor-causing bacteria on the skin may lead to severe body odor. Therefore, the generation of biofilms in fabrics that come in direct contact with the skin must be suppressed.

In this experiment, the extent to which the bacteria adhered to the fabric was quantitatively confirmed by determining the CFU of the bacteria attached to the fabric surface. Moreover, the amount of biofilm generated on the fabric surface was determined through fluorescent dye staining. Specifically, our study examined the growth of *E. coli, P. aeruginosa, S. epidermidis, C. xerosis*, and *M. luteus*. First, the CFU was determined through a comparative analysis of untreated and rGO/Cu NP-treated cotton fabrics. The degree of adhesion of *P. aeruginosa* and *S. epidermidis* was decreased by approximately 40% in the rGO/Cu fabrics. However, in the rGO/Cu fabrics, the value for *E. coli* decreased by 81.4% from 1.4 × 10^6^ CFU/mL to 2.6 × 10^5^ CFU/mL, whereas that of *C. xerosis* decreased by 82.9% from 7.2 × 10^4^ CFU/mL to 1.2 × 10^4^ CFU/mL. Notably, the value for *M. luteus* decreased by 99.2% from 1.2 × 10^7^ CFU/mL to 1.0 × 10^5^ CFU/mL. Overall, the degree of adhesion of various bacteria was significantly decreased by coating rGO/Cu on the fabric. Next, the degree of inhibition of biofilm formation was confirmed by dyeing the biofilm formed on the fabric. As shown in Fig. 5, high-intensity green fluorescence could be observed on the untreated cotton fabric. In contrast, in the rGO/Cu fabrics, an extremely low fluorescence signal was observed for all types of bacteria. These findings demonstrated that the rGO/Cu fabrics could sufficiently suppress biofilm formation. Therefore, the rGO/Cu fabrics still eradicate bacteria and their biofilm even after being aerated and re-oxidized sufficiently through rGO/Cu coating.

In our previous study, the antibacterial effect was observed for a longer period of time. First, we synthesized Cu NPs on multi-walled carbon nanotubes (MWCNTs) and confirmed that their antibacterial effect was maintained for up to 48 h^21^. In another previous study, we fabricated Ag/Cu/GO and confirmed that their antibacterial effect was maintained for up to 72 h in vitro. Additionally, in an in vivo experiment, a *P. aeruginosa* biofilm was treated with 1 mg/mL of Ag/Cu/GO and observed for 7 days. The results confirmed that the biofilm was sufficiently removed and the wound healed well. More importantly, the produced Ag/Cu/GO was able to maintain antibacterial and antibiofilm effects for 7 days^22^. Although this study confirmed the antibacterial effect of the rGO/Cu fabric for up to 24 h, our previous findings suggest that this antibacterial effect is likely to last longer. Generally, biofilm formation takes from 12 to 72 h depending on the species^49–51^. Furthermore, given that the developed rGO/Cu fabric is intended to be used as a disposable fabric, the fabric can be exchanged periodically before the bacteria grow back. Therefore, the material developed herein is suitable for use as a disposable fabric with long-term antibacterial and anti-biofilm effects.

## Conclusions

Fabrics are an essential component of modern life. However, cotton fabrics are vulnerable to infection by various pathogenic microorganisms such as bacteria, fungi, and viruses owing to their excellent hygroscopicity. Infection with microorganisms such as bacteria or viruses may lead to odor issues and threaten human health. Therefore, the development of biocompatible antimicrobial fabrics is essential. In this study, antibacterial fabrics were developed using rGO/Cu nanocomposites. First, by coating rGO/Cu nanocomposites on the fabrics, the surface roughness was changed, and the hydrophilic fabrics became hydrophobic. This created an environment in which the bacteria could not adhere to the fabrics, and the formation of biofilms was thus suppressed. Additionally, rGO/Cu nanocomposites coated on the antibacterial fabric released copper ions through the oxidation–reduction process, thus exhibiting an effective antibacterial effect against bacteria. Therefore, our rGO/Cu fabric can prevent wound infection and unpleasant odors caused by sweat. These properties are maintained even when the rGO/Cu fabric is aerated sufficiently. Furthermore, the antibacterial fabrics developed in this study were confirmed to be biocompatible, as there were no signs of cytotoxicity to human and mouse skin cells after up to 24 h of exposure. Nevertheless, additional research is needed to produce reusable fabrics.

## Supplementary Information

Supplementary Figures.

## Data Availability

The datasets used and/or analyzed during the current study are available from the corresponding author upon reasonable request.
