# Supplementary material for: Antibacterial and biofilm-inhibiting cotton fabrics decorated with copper nanoparticles grown on graphene nanosheets
Source: Sci Rep. 2023 Jul 24;13:11947. doi: 10.1038/s41598-023-38723-4 (PMC10366191; doi:10.1038/s41598-023-38723-4)
Supplement: Supplementary file 1 — Supplementary Figures. [file 41598_2023_38723_MOESM1_ESM.docx]

**Supplementary Information**

Antibacterial and biofilm-inhibiting cotton fabrics decorated with copper nanoparticles grown on graphene nanosheets

Jiwon Kim^1-2^, Seung Hyun Kang^3^, Yonghyun Choi^1,4^, Wonjae Lee^1^, Nayeong Kim^1^, Masayoshi Tanaka^5^, Shink Hyuk Kang^*,3^, Jonghoon Choi^*,1-2,4^

^1^School of Integrative Engineering, Chung-Ang University, Seoul 06974, Republic of Korea

^2^Department of Chemical and Biomolecular Engineering, University of Pennsylvania, Philadelphia, PA 19104, USA

^3^Departments of Plastic and Reconstructive Surgery, Chung-Ang University Hospital, Chung-Ang University College of Medicine, Seoul 06973, Republic of Korea

^4^Feynman Institute of Technology, Nanomedicine Corporation, Seoul 06974, Republic of Korea

^5^Department of Chemical Science and Engineering, Tokyo Institute of Technology, 4259 Nagatsuta-cho, Midori-ku, Yokohama-shi, Kanagawa 226-8503, Japan

*Correspondence and request for materials should be addressed to:

kangshinhyeok@cau.ac.kr (S.H. Kang) and nanomed@cau.ac.kr (J. Choi)

**Table of Contents**

Figure S1. Hydrophobicity test of initial rGO/Cu fabric

Figure S2. *In vitro* cytotoxicity test of HDF and NIH/3T3 cultured in the leachate of raw fabric and rGO/Cu fabric


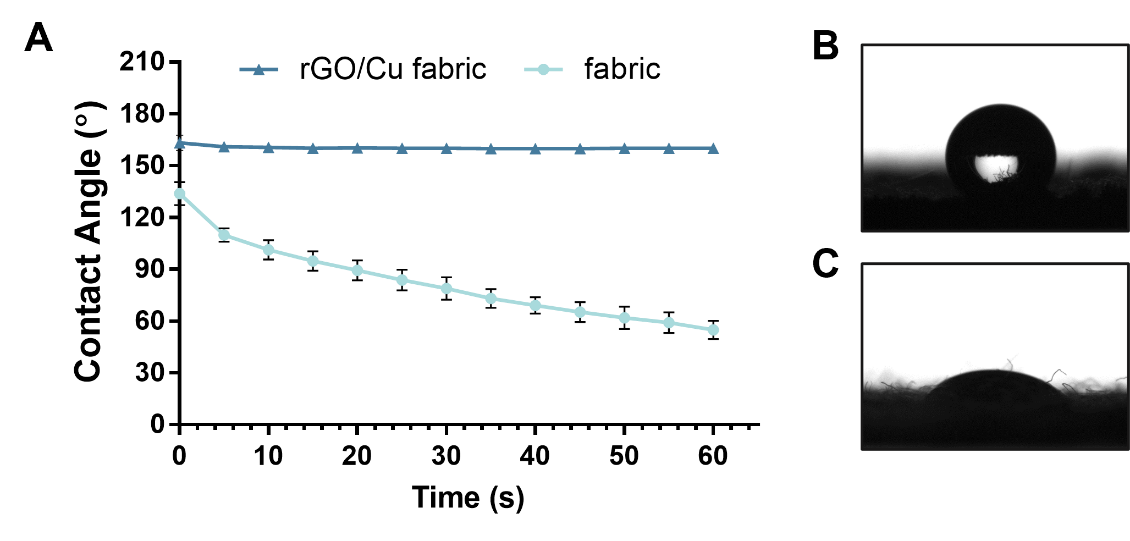


**Figure S1.** Hydrophobicity test of initial rGO/Cu fabric. (A) Contact angles of fabric and rGO/Cu fabric. Images of 5 μL DW droplet on (B) rGO/Cu fabric and (C) fabric at different intervals within 60 seconds.


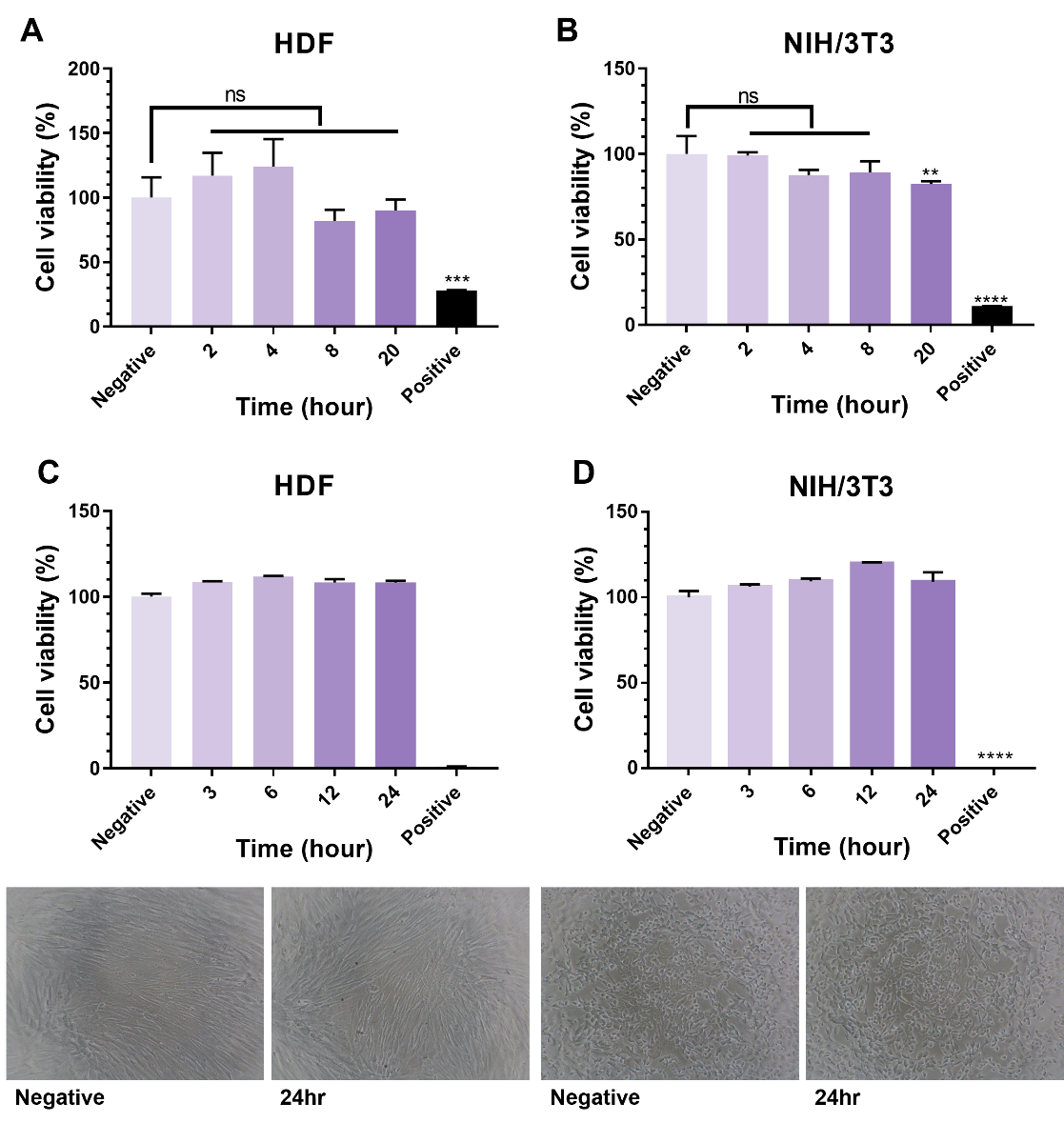


**Figure S2.** *In vitro* cytotoxicity test of (A,C) HDF and (B,D) NIH/3T3 cultured in the leachate of raw fabric and rGO/Cu fabric for 24 h. (A,B) is the cell viability immediately after rGO/Cu fabric fabrication, and (C,D) is the cell viability of rGO/Cu fabric that is aerated and re-oxidized. The data are presented as mean ± SD (n=3).
